# Supplementary material for: Evaluation of Tumor Grade and Proliferation Indices before and after Short-Course Anti-Inflammatory Prednisone Therapy in Canine Cutaneous Mast Cell Tumors: A Pilot Study
Source: Vet Sci. 2022 Jun 7;9(6):277. doi: 10.3390/vetsci9060277 (PMC9227510; doi:10.3390/vetsci9060277)
Supplement: Supplementary file 1 [file vetsci-09-00277-s001.zip › vetsci-1734668-supplementary.pdf]

Table S1. Paired tumor grades, mitotic counts, and proliferation indices.

	Pre-treatment					Post-treatment				
**Patient #**	Mitotic Count	Grade	AgNOR	Ki67	AgNOR x Ki67	Mitotic Count	Grade	AgNOR	Ki67	AgNOR x Ki67
1	2	G2/LG	1.5	6.6	9.9	0	G2/LG	1.35	4.4	5.9
2	0	G2/LG	1.61	4.6	7.4	1	G2/LG	1.63	5.4	8.8
3	8	G2/HG	2.6	32.4	84.2	25	G3/HG	2.8	35	98
4	2	G2/LG	1.47	11.4	16.8	1	G2/LG	1.64	1.6	2.6
5	3	G2/LG	3.07	17.2	52.8	4	G2/LG	2.2	15.8	34.8
6	2	G2/LG	1.13	1.8	2	2	G2/LG	1.36	4	5.4
7	0	G1/LG	1.1	4.4	4.8	2	G2/LG	1.06	12.6	13.4
8	1	G2/LG	2.8	5	14	2	G2/LG	1.4	2.8	3.9
9	3	G2/LG	1.7	12.6	21.4	0	G2/LG	1.5	8	12
10	1	G2/LG	1.97	8.2	16.2	0	G2/LG	0.93	2.2	2.1
11	2	G2/LG	1.5	6.8	10.2	2	G2/LG	1.13	6.4	7.2
